# Supplementary material for: A yeast phenomic model for the influence of Warburg metabolism on genetic buffering of doxorubicin
Source: Cancer Metab. 2019 Oct 23;7:9. doi: 10.1186/s40170-019-0201-3 (PMC6806529; doi:10.1186/s40170-019-0201-3)
Supplement: Supplementary file 9 — Additional file 9. GO term-specific heatmaps for REMc/GTF-enriched clusters. GO term-specific heatmaps for significant GO process terms were generated as described in methods and Figs. 3 and 4. Any related child terms are presented in subsequent pages of the parent file name. GO terms with more than 100 children, with 2 or fewer genes annotated to the term, or a file size over 300KB are not shown. All heatmaps are generated with the same layout (see Figs. 3 and 4). [file 40170_2019_201_MOESM9_ESM.bz2 › Additional_File9_GOTermHeatmaps/Additional_File9_GOTermHeatmap/telomere_localization.pdf]

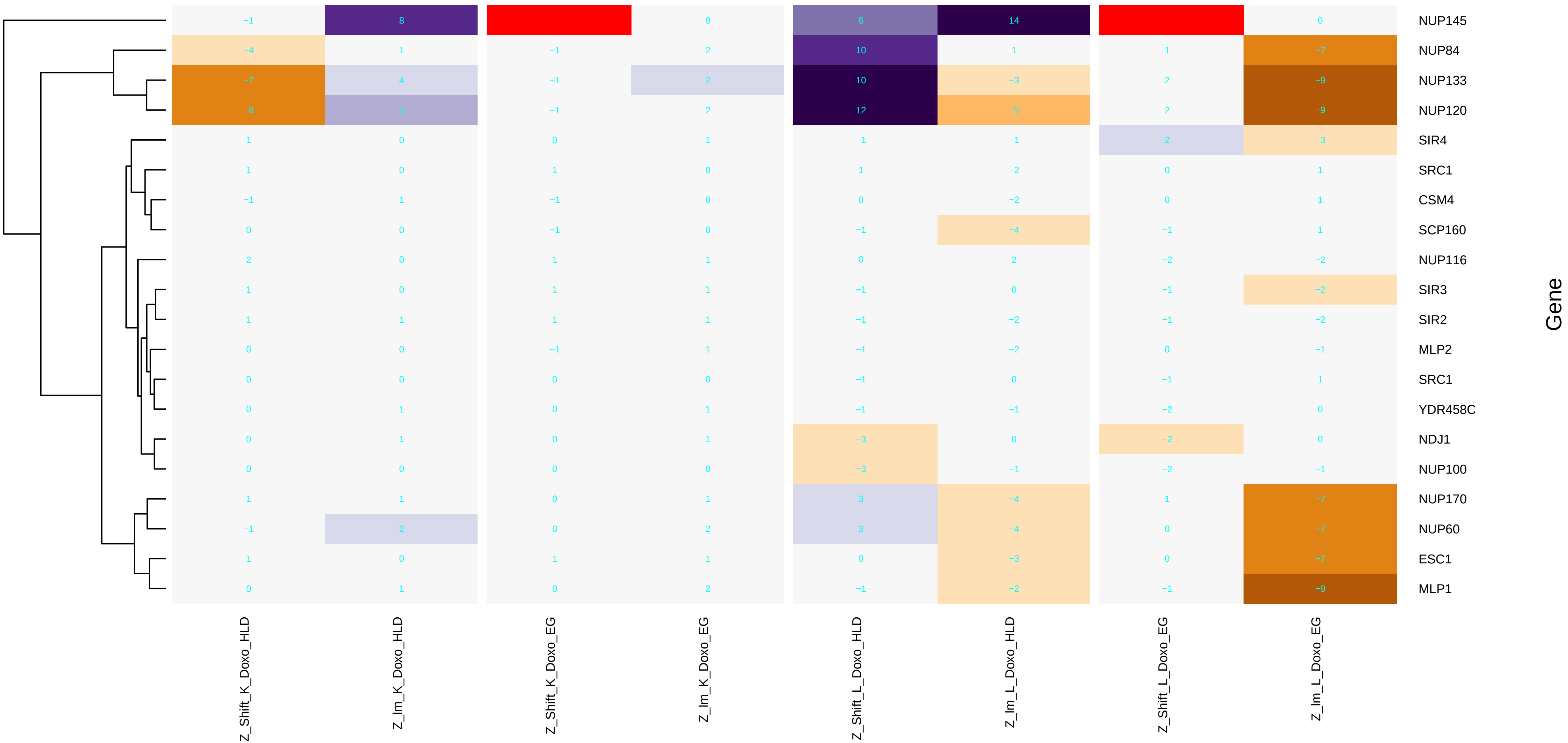
NUP145 −1 8 0 6 14 0
NUP84 −4 1 −1 2 10 1 1 −7
NUP133 −7 4 −1 2 10 −3 2 −9
NUP120 −8 5 −1 2 12 −5 2 −9
SIR4 1 0 0 1 −1 −1 2 −3
SRC1 1 0 1 0 1 −2 0 1
CSM4 −1 1 −1 0 0 −2 0 1
SCP160 0 0 −1 0 −1 −4 −1 1
NUP116 2 0 1 1 0 2 −2 −2
SIR3 1 0 1 1 −1 0 −1 −2
SIR2 1 1 1 1 −1 −2 −1 −2
MLP2 0 0 −1 1 −1 −2 0 −1
SRC1 0 0 0 0 −1 0 −1 1
YDR458C 0 1 0 1 −1 −1 −2 0
NDJ1 0 1 0 1 −3 0 −2 0
NUP100 0 0 0 0 −3 −1 −2 −1
NUP170 1 1 0 1 3 −4 1 −7
NUP60 −1 2 0 2 3 −4 0 −7
ESC1 1 0 1 1 0 −3 0 −7
MLP1 0 1 0 2 −1 −2 −1 −9
Gene
Z_Shift_K_Doxo_HLD
Z_Im_K_Doxo_HLD
Z_Shift_K_Doxo_EG
Z_Im_K_Doxo_EG
Z_Shift_L_Doxo_HLD
Z_Im_L_Doxo_HLD
Z_Shift_L_Doxo_EG
Z_Im_L_Doxo_EG
